# Safety and efficacy assessment of allogeneic human dental pulp stem cells to treat patients with severe COVID-19: structured summary of a study protocol for a randomized controlled trial (Phase I / II)

**DOI:** 10.1186/s13063-020-04380-5

**Published:** 2020-06-12

**Authors:** Qingsong Ye, Hua Wang, Xia Xia, Chenliang Zhou, Zhiming Liu, Zun-en Xia, Zhan Zhang, Yang Zhao, Jun Yehenala, Si Wang, Gangqiao Zhou, Ke Hu, Bin Wu, Chu-Tse Wu, Songling Wang, Yan He

**Affiliations:** 1 Renmin Hospital of Wuhan University, Wuhan, 430060 Hubei China; 2 Beijing Institute of Radiation Medicine, Beijing, 100850 China; 3 Beijing SH Biotechnology Co., Ltd., Beijing, 100070 China; 4 Tianjin Fopcells Pharmaceutical Technology Co., Ltd, Tianjin, 300074 China; 5 School of Stomatology, Capital Medical University, Beijing, 100006 China; 6 Tianyou Hospital, Wuhan University of Science and Technology, Wuhan, 430064 Hubei China; 7 Utooth Biological Technology Co., Ltd., Hubei Branch, Wuhan, 430060 Hubei China

**Keywords:** COVID-19; randomised controlled trial; protocol; human dental pulp stem cells; dental stem cell banking

## Abstract

**Objectives:**

To assess the safety and therapeutic effects of allogeneic human dental pulp stem cells (DPSCs) in treating severe pneumonia caused by COVID-19.

**Trial design:**

This is a single centre, two arm ratio 1:1, triple blinded, randomized, placebo-controlled, parallel group, clinical trial.

**Participants:**

Twenty serious COVID-19 cases will be enrolled in the trial from April 6th to December 31st 2020. Inclusion Criteria: hospitalised patients at Renmin Hospital of Wuhan University satisfy all criteria as below:

1. Adults aged 18-65 years;
2. Voluntarily participate in this clinical trial and sign the “informed consent form” or have consent from a legal representative.
3. Diagnosed with severe pneumonia of COVID-19: nucleic acid test SARS-CoV-2 positive; respiratory distress (respiratory rate > 30 times / min); hypoxia (resting oxygen saturation < 93% or arterial partial pressure of oxygen / oxygen concentration < 300 mmHg).
4. COVID-19 featured lung lesions in chest X-ray image.

Exclusion Criteria: Patients will be excluded from the study if they meet any of the following criteria.

1. Patients have received other experimental treatment for COVID-19 within the last 30 days;
2. Patients have severe liver condition (e.g., Child Pugh score >=C or AST> 5 times of the upper limit);
3. Patients with severe renal insufficiency (estimated glomerular filtration rate <=30mL / min/1.73 m^2^) or patients receiving continuous renal replacement therapy, hemodialysis, peritoneal dialysis;
4. Patients who are co-infected with HIV, hepatitis B, tuberculosis, influenza virus, adenovirus or other respiratory infection viruses;
5. Female patients who have no sexual protection in the last 30 days prior to the screening assessment;
6. Pregnant or lactating women or women using estrogen contraception;
7. Patients who are planning to become pregnant during the study period or within 6 months after the end of the study period;
8. Other conditions that the researchers consider not suitable for participating in this clinical trial.

**Intervention and comparator:**

There will be two study groups: experimental and control. Both will receive all necessary routine treatment for COVID-19.

The experimental group will receive an intravenous injection of dental pulp stem cells suspension (3.0x10^7^ human DPSCs in 30ml saline solution) on day 1, 4 and 7;

The control group will receive an equal amount of saline (placebo) on the same days.

Clinical and laboratory observations will be performed for analysis during a period of 28 days for each case since the commencement of the study.

**Main outcomes:**

1. Primary outcome

The primary outcome is Time To Clinical Improvement (TTCI). By definition, TTCI is the time (days) it takes to downgrade two levels from the following six ordered grades [(grade 1) discharge to (grade 6) death] in the clinical state of admission to the start of study treatments (hDPSCs or placebo).

Six grades of ordered variables:

**Table:** 

Grade	Description
Grade 1:	Discharged of patient;
Grade 2:	Hospitalized without oxygen supplement;
Grade 3:	Hospitalized, oxygen supplement is required, but NIV / HFNC is not required;
Grade 4:	Hospitalized in intensive care unit, and NIV / HFNC treatment is required;
Grade 5:	Hospitalized in intensive care unit, requiring ECMO and/or IMV;
Grade 6:	Death.

Abbreviations: NIV, non-invasive mechanical ventilation; HFNC, high-flow nasal catheter; IMV, invasive mechanical ventilation.

2. Secondary outcomes

2.1 vital signs: heart rate, blood pressure (systolic blood pressure, diastolic blood pressure). During the screening period, hospitalization every day (additional time points of D1, D4, D7 30min before injection, 2h ± 30min, 24h ± 30min after the injection) and follow-up period D90 ± 3 days.

2.2 Laboratory examinations: during the screening period, 30 minutes before D1, D4, D7 infusion, 2h ± 30min, 24h ± 30min after the end of infusion, D10, D14, D28 during hospitalization or discharge day and follow-up period D90 ± 3 days.

2.3 Blood routine: white blood cells, neutrophils, lymphocytes, monocytes, eosinophils, basophils, neutrophils, lymphocytes, monocytes, eosinophils Acidic granulocyte count, basophil count, red blood cell, hemoglobin, hematocrit, average volume of red blood cells, average red blood cell Hb content, average red blood cell Hb concentration, RDW standard deviation, RDW coefficient of variation, platelet count, platelet specific platelet average Volume, platelet distribution width,% of large platelets;

2.4 Liver and kidney function tests: alanine aminotransferase, aspartate aminotransferase, alkaline phosphatase, γ-glutamyl transferase, prealbumin, total protein, albumin, globulin, white / globule ratio , Total bilirubin, direct bilirubin, cholinesterase, urea, creatinine, total carbon dioxide, uric acid glucose, potassium, sodium, chlorine, calcium, corrected calcium, magnesium, phosphorus, calcium and phosphorus product, anion gap, penetration Pressure, total cholesterol, triacylglycerol, high density lipoprotein cholesterol, Low density lipoprotein cholesterol, lipoprotein a, creatine kinase, lactate dehydrogenase, estimated glomerular filtration rate.

2.5 Inflammation indicators: hypersensitive C-reactive protein, serum amyloid (SAA);

2.6 Infectious disease testing: Hepatitis B (HBsAg, HBsAb, HBeAg, HBeAb, HBcAb), Hepatitis C (Anti-HCV), AIDS (HIVcombin), syphilis (Anti-TP), cytomegalovirus CMV-IgM, cytomegalovirus CMV-IgG; only during the screening period and follow-up period D90 ± 3.

2.7 Immunological testing:

Collect peripheral blood to detect the phenotype of T lymphocyte, B lymphocyte, natural killer cell, Macrophage and neutrophil by using flow cytometry.

Collect peripheral blood to detect the gene profile of mononuclear cells by using single-cell analyses.

Collect peripheral blood serum to detect various immunoglobulin changes: IgA, IgG, IgM, total IgE;

Collect peripheral blood serum to explore the changes of cytokines, Th1 cytokines (IL-1 β, IL-2, TNF-a, ITN-γ), Th2 cytokines (IL-4, IL-6, IL -10).

2.8 Pregnancy test: blood β-HCG, female subjects before menopause are examined during the screening period and follow-up period D90 ± 3.

2.9 Urine routine: color, clarity, urine sugar, bilirubin, ketone bodies, specific gravity, pH, urobilinogen, nitrite, protein, occult blood, leukocyte enzymes, red blood cells, white blood cells, epithelial cells, non-squamous epithelial cells , Transparent cast, pathological cast, crystal, fungus;

2.10 Stool Routine: color, traits, white blood cells, red blood cells, fat globules, eggs of parasites, fungi, occult blood (chemical method), occult blood (immune method), transferrin (2h ± 30min after the injection and not detected after discharge).

**Randomization:**

Block randomization method will be applied by computer to allocate the participants into experimental and control groups. The random ratio is 1:1.

**Blinding (masking):**

Participants, outcomes assessors and investigators (including personnel in laboratory and imaging department who issue the sample report or image observations) will be blinded.

Injections of cell suspension and saline will be coded in accordance with the patient’s randomisation group.

The blind strategy is kept by an investigator who does not deliver the medical care or assess primary outcome results.

**Numbers to be randomized (sample size):**

Twenty participants will be randomized to the experimental and control groups (10 per group).

**Trial Status:**

Protocol version number, hDPSC-CoVID-2019-02-2020 Version 2.0, March 13, 2020.

Patients screening commenced on 16^th^ April and an estimated date of the recruitment of the final participants will be around end of July. .

**Trial registration:**

Registration:

World Health Organization Trial Registry: ChiCTR2000031319; March 27,2020.

ClinicalTrials.gov Identifier: NCT04336254; April 7, 2020

Other Study ID Numbers: hDPSC-CoVID-2019-02-2020

**Full protocol:**

The full protocol is attached as an additional file, accessible from the Trials website (Additional file 1). In the interest in expediting dissemination of this material, the familiar formatting has been eliminated; this Letter serves as a summary of the key elements of the full protocol.

## Supplementary information

**Additional file 1.** Full study protocol.

## Data Availability

The data will be stored in a repository managed by a third party called Clinflash, a professional data management service for clinical studies (weblink: https://edc.clinflash.net/shbio). Member investigators of the trial will have access to the final trial dataset, depending on level of need-to-know.

