# Supplementary material for: Safety and efficacy assessment of allogeneic human dental pulp stem cells to treat patients with severe COVID-19: structured summary of a study protocol for a randomized controlled trial (Phase I / II)
Source: Trials. 2020 Jun 12;21:520. doi: 10.1186/s13063-020-04380-5 (PMC7290137; doi:10.1186/s13063-020-04380-5)
Supplement: Supplementary file 1 — Additional file 1. Full study protocol. [file 13063_2020_4380_MOESM1_ESM.pdf]

临床研究方案

异体人牙髓间充质干细胞治疗 COVID-19 重症肺炎的安全性和有效性研究

方　案　号：hDPSC-CoVID-2019-02-2020

版　　　本： V 2.0 版，2020 年 3 月 13 日

项目承担单位: 武汉大学人民医院

承担单位地址：湖北省武汉市武昌区张之洞路(原紫阳路)99 号解放路 238 号

主要研究者：叶青松、周晨亮、吴祖泽、王松灵、张　旃、王　华、
夏　侠、贺燕、刘志明、叶赫娜拉俊、夏尊恩、贺嘉

项目承担科室：武汉大学人民医院再生医学中心、东院重症医学科

项目合作单位： 北京三有利和泽生物科技有限公司、湖北优牙生物科技有限公司、
首都医科大学、北京吴祖泽科技发展基金会、
方普医药科技(天津)有限公司

保密声明

本临床研究方案内含商业机密并且该商业信息享有特权，方案中所包含的有关制剂所有信息的所有权归北京三有利和泽生物科技有限公司和首都医科大学，未经法律许可不得公开。因此，仅提供给研究者、合作研究者、伦理委员会和监督管理部门等相关的机构审阅。未经项目承担单位书面批准的情况下，各合作单位和个人严禁将本研究任何信息透露给与本研究无关的第三方。同时，在未得到合作单位书面的批准情况下，除了在与可能参加本研究的受试者签署知情同意时，向其做必要的解释外，严禁将任何信息告知与本研究无关的第三方。

主要研究者：

主要研究者	武汉大学人民医院　　叶青松　、周晨亮		
地址	湖北省武汉市武昌区张之洞路(原紫阳路)99 号解放路 238 号		
邮编	430060	电话	15858242516、18171234829

项目名称：异体人牙髓间充质干细胞治疗 COVID-19 重症肺炎的安全性和有效性研究

方　案　号： hDPSC-CoVID-2019-02-2020

版　　本： V 2.0 版，2020 年 3 月 13 日

研究者名单：

	单位名称	科室/职务	姓名
01	武汉大学人民医院	国家特聘专家、再生医学中心主任	叶青松
02	武汉大学人民医院	东院区重症医学科主任	周晨亮
03	军事科学院军事医学研究院	中科院院士<br>首席科学家、学术委员会主任	吴祖泽
04	首都医科大学	中科院院士、副校长	王松灵
05	武汉大学人民医院	呼吸重症二科主任	张旃
06	军事科学院军事医学研究院	副主任<br>合作专家	王华
07	北京三有利和泽生物科技有限公司	临床医学部主任	夏侠
08	湖北优牙生物科技有限公司	牙髓干细胞特聘专家	贺燕
09	武汉大学人民医院	再生医学中心	刘志明
10	武汉人民医院	东院区重症医学科护士	叶赫娜拉俊
11	武汉人民医院	检验科	夏尊恩
12	北京三有利和泽生物科技有限公司	临床医学部	贺嘉

研究者声明

我承诺：

1. 遵守本临床研究方案（“异体人牙髓间充质干细胞治疗 COVID-19 重症肺炎的安全性和有效性研究 ”），方案号：hDPSC-CoVID-2019-02-2020。

2. 严格按照赫尔辛基宣言、中国现行法规、以及临床研究方案的要求进行本次临床研究。

3. 规范记录、保存临床研究原始数据。

4. 允许监管部门对本临床研究项目进行检查。

我已全部阅读了临床研究方案，包括以上的声明，我同意以上全部内容，并将对以上全部内容保密。

项目承担与申办单位：武汉大学人民医院

主要研究者（签字）：________________

日　期：________年____月____日

合作研究者声明

我承诺：

遵守本临床研究方案（“异体人牙髓间充质干细胞治疗 COVID-19 重症肺炎的安全性和有效性研究 ”），方案号：hDPSC-CoVID-2019-02-2020。

严格按照赫尔辛基宣言、中国现行法规、以及临床研究方案的要求进行本次临床研究细胞制备，规范制备、质检、出库、运输记录、保存细胞制备相关原始数据，允许监管部门对该项目进行检查。

我已同意以上声明的全部内容，并将对以上全部内容保密。

细胞制备机构：北京三有利和泽生物科技有限公司

制剂负责人（签字）：_________________

日　　期：　　　________年____月____日

合作研究者声明

我承诺：

遵守本临床研究方案（“异体人牙髓间充质干细胞治疗 COVID-19 重症肺炎的安全性和有效性研究 ”），方案号：hDPSC-CoVID-2019-02-2020。

严格按照赫尔辛基宣言、中国现行法规、以及临床研究方案的要求进行本次临床研究细胞制剂制备，规范制备、质检、出库、运输记录、保存细胞制备相关原始数据，允许监管部门对该项目进行检查。

我已同意以上声明的全部内容，并将对以上全部内容保密。

细胞制剂制备机构：湖北优牙生物科技有限公司

制剂负责人（签字）：________________

日　期：　　________年____月____日

1 方案摘要

**研究方案号**	hDPSC-CoVID-2019-02-2020
**方案名称**	异体人牙髓间充质干细胞治疗 COVID-19 重症肺炎的安全性和有效性研究
**版本号/日期**	V2.0 版，2020 年 3 月 13 日
**研究类别**	临床探索性研究
**研究制剂**	人牙髓间充质干细胞（hDPSC）
**适应症**	新型冠状病毒所致重症肺炎
**研究目的**	评价应用人牙髓间充质干细胞治疗新型冠状病毒所致重症肺炎的安全性和有效性；为探索应用人牙髓间充质干细胞治疗新型冠状病毒所致重症肺炎，降低死亡率，改善临床预后提供新的治疗方案。
**病例总数**	20 例
**研究中心数**	1 个
**研究期限**	2020 年 3 月-2020 年 6 月完成入组，2021 年 3 月完成总结分析。
**研究设计**	本研究为单中心随机、单盲、安慰剂对照研究。临床诊断为新型冠状病毒所致重症肺炎的受试者签署知情同意书并符合纳入标准且不符合排除标准后，所有受试者均接受新冠肺炎常规治疗，并于常规治疗同时接受 hDPSC 细胞或安慰剂静脉输注治疗。所有受试者接受研究治疗后观察至 D28 或出院，出院后持续随访收集受试者临床安全和有效性观察指标直至 D90 结束研究。<br>由于入选患者均为重症，常规治疗将根据《新型冠状病毒肺炎诊疗方案（试行第七版）》的治疗原则，对受试者采用相同/相似的临床方案，可以保证组间常规治疗的一致性。如无特殊原因，除本方案确定的常规治疗和干细胞治疗外，试验过程中不再使用其他药物进行治疗。<br>**（1）随机对照研究设计**<br>本研究共入组 20 例受试者，采用随机、安慰剂对照设计，将受试者随机分配至常规治疗+安慰剂组和常规治疗+hDPSC 细胞治疗组，每组分别为 10 例。常规治疗+安慰剂组：该组受试者接受新冠肺炎常规治疗同

	时分别于 D1、D4、D7 接受静脉输注 30mL 安慰剂，采用一次性输血器进行静脉输注；常规治疗+hDPSC 细胞治疗组：该组受试者在接受新冠肺炎常规治疗的同时，分别于 D1、D4 和 D7 接受 hDPSC 输注治疗，采用一次行输血器进行输注，输注的细胞剂量为 3.0×10^7 hDPSC 细胞/人，输注周期为一周，每次输注间隔 2 天。<br>为控制细胞治疗试验风险，常规治疗+hDPSC 细胞治疗组采取分步实施，首例受试者给药疗程结束（D1-D7）且研究者根据安全性评价指标判断为给药安全时，再进行本组后续受试者的入组治疗；如出现 1 例受试者发生很可能或肯定与试验药物相关的严重不良事件，则终止本试验。<br>**（2）细胞治疗剂量设计**<br>根据临床前研究结果和 ClinicalTrials.gov 注册项目“间充质干细胞治疗 2019 新型冠状病毒感染的肺炎患者的安全性和有效性”的给药设计（每例受试者每疗程给予 1.5-3.0 $\times10^6$ 细胞/ kg），本项目每例受试者每次输注细胞剂量为：约 3.0×10^7 细胞/人，每 3 次细胞输注为 1 个治疗疗程（按每例受试者每疗程 1.5×10^6 细胞/ kg 计算）。<br>**（3）盲法设计**<br>采取单盲设计，对受试者、临床观察和统计分析人员设盲。
**目标受试者人群**	**纳入标准：**受试者必须满足以下所有标准，方可纳入本研究：<br>（1） 年龄 18-65 周岁，性别不限；<br>（2） 自愿参加本临床研究并提供《知情同意书》；<br>（3） 诊断为重型新冠肺炎：呼吸窘迫，RR≥30 次/分；静息状态下，指氧饱和度≤93%；动脉血氧分压/吸氧浓度≤300mmHg；新冠病毒（CoVID-19）核酸检测为阳性。<br>（4） 胸部影像检查证实肺脏受累。<br>**排除标准：**符合下列任意一项的受试者，将被排除在此项研究之外：<br>（1） 在筛选评估之前的 30 天内接受任何针对 COVID-19 的临床试验

	药物治疗者；<br>（2） 严重肝病（例如 Child Pugh 分数≥C 或 AST>上限的 5 倍）；<br>（3） 已知严重肾功能不全者（估计肾小球滤过率≤30mL/min/1.73 m2）或接受连续性肾脏替代治疗，血液透析，腹膜透析的患者；<br>（4） HIV，乙肝，结核，流感病毒，腺病毒和其他呼吸道感染病毒的共同感染；<br>（5） 研究筛选前一个月之内没有保护措施的性生活的女性患者；<br>（6） 妊娠、哺乳期妇女或应用雌性激素避孕的女性；<br>（7） 本人或配偶计划在研究期间和研究结束后 6 个月内妊娠的患者；<br>（8） 研究者认为其他不适宜参加的情况。<br>**退出/脱落标准：**<br>（1） 受试者在研究过程中发生了严重不良事件，研究者认为不能再继续进行研究；<br>（2） 受试者依从性差，不能按时完成随访；<br>（3） 受试者不愿继续进行临床研究，向研究者提出退出；<br>（4） 失访。
**研究终止**	**终止标准：**临床研究中一旦发现以下任一问题，则终止该研究：<br>（1） 研究中发生严重安全性问题（如出现严重不良反应者或出现严重并发症或病情迅速恶化者）；<br>（2） 研究中发现效果较差，甚至无效，不具有临床价值，继续进行下去会延误受试者治疗；<br>（3） 研究中发现临床研究方案制订有重大失误，或方案实施有重大偏差，再继续下去难以评价。
**研究步骤**	**（1）筛选入组：**受试者签署知情同意书后，在研究机构的综合诊疗中心进行各项入组前筛选检查，符合纳入标准且不符合排除标准的受试者入组。入组后，按照随机原则分配到不同组别。<br>**（2）给药治疗：**研究给药周期为1周，每次治疗间隔2天。<br>▪ **新冠肺炎（NCP）常规治疗**+安慰剂：在对症治疗的基础上，积极防

	治并发症，治疗基础疾病，预防继发感染，及时进行器官功能支持；呼吸支持；循环支持，同时分别于D1、D4、D7接受给予30mL安慰剂静脉输注治疗，输注时采用一次性输血器进行输注。<br>▪ **新冠肺炎（NCP）常规治疗+干细胞静脉输注治疗**：受试者在接受新冠肺炎（NCP）常规治疗（同上）的同时，分别于D1、D4和D7给予hDPSC细胞静脉输注治疗，输注采用一次性输血器进行输注，细胞输注剂量为3.0×10^{7} hDPSC细胞/人，<br>**（3）治疗观察（D1-D28或出院日）**：所有受试者在此期间均接受每日生命体征、临床检测指标检查和不良事件收集，并于D1、D4、D7、D10、D14、D28或出院日接受临床安全性指标和有效性指标评价，所有受试者均增加输注给药后2h±30min和24±30min的安全性评估，包括生命体征检查和实验室安全性指标检查。<br>**（5）随访**（D90±3）：受试者治疗出院后于D90±3进行随访，收集临床疗效和免疫学评价指标，结束研究。
**安全性评估**	①生命体征：心率、血压（收缩压、舒张压）。在筛选期、住院期间每日（另外增加D1、D4、D7输注前30min、输注结束后2h±30min、24h±30min）和随访期D90±3进行。<br>②实验室检查：在筛选期、D1、D4、D7输注前30min、输注结束后2h±30min、24h±30min、住院期间D10、D14、D28或出院日和随访期D90±3进行。<br>▪ 血常规：白细胞、红细胞、血红蛋白、红细胞压积、平均红细胞体积、平均血红蛋白量、平均血红蛋白浓度、血小板计数、血小板平均体积、血小板压积、中性粒细胞、淋巴细胞、单核细胞、嗜酸细胞、嗜碱细胞、中性粒细胞绝对值、淋巴细胞绝对值、单核细胞绝对值、嗜酸细胞绝对值、嗜碱细胞绝对值、红细胞分布宽度SD、红细胞分布宽度CV、大血小板比率、血小板分布宽度；<br>▪ 肝肾功能：丙氨酸氨基转移酶、天门冬氨酸氨基转移酶、总蛋白、白蛋白、球蛋白、白球比、总胆红素、直接胆红素、间接胆红素、碱性磷酸酶、肌酐、γ-谷氨酰基转移酶、乳酸脱氢酶、肌酸激酶、尿素、

	尿酸、总二氧化碳、葡萄糖；<br>▪ 炎症指标：超敏C反应蛋白、血清淀粉样蛋白（SAA）；<br>▪ 传染病检测：乙肝（HBsAg、HBsAb、HBeAg、HBeAb、HBcAb）、丙肝（Anti-HCV）、艾滋病（HIVcombin）、梅毒（Anti-TP）、巨细胞病毒CMV-IgM、巨细胞病毒CMV-IgG；仅在筛选期、随访期D90±3检查。<br>▪ 免疫学检测：<br>✓ 收集外周抗凝血，检测淋巴细胞亚群变化：T淋巴细胞（CD3、CD4、CD8）、B淋巴细胞（CD19）、NK细胞（CD16、CD56）；<br>✓ 收集外周血血清，检测各种免疫球蛋白变化：IgA、IgG、IgM、总IgE；<br>✓ 收集外周血血清，探索细胞因子的变化规律，Th1类细胞因子（IL-1β、IL-2、TNF-a、ITN-γ），Th2类细胞因子（IL-4、IL-6、IL-10）。<br>▪ 妊娠试验：血β-HCG，绝经期前的女性受试者在筛选期、随访期D90±3检查。<br>▪ 尿常规：PH值、比重、蛋白质、酮体、胆红素、尿胆素原、亚硝酸盐、白细胞、红细胞、尿糖、尿沉渣镜检；<br>▪ 便常规：颜色、性状、白细胞、红细胞、脂肪球、虫卵、真菌、潜血（出院后不检测）<br>③不良事件和严重不良事件：研究期间及时记录不良事件和严重不良事件发生情况，不良事件程度按NCI CTCAE v5.0分级标准判定。
有效性评估	1.主要疗效指标<br>临床改善时间（TTCI）时间范围：直至28天或出院<br>TTCI的定义是开始研究治疗（阳性药或安慰剂）从入院临床状态的以下六类有序等级变量【（1）出院到（6）死亡】中下降两个等级所经历的时间（天）。<br>六类有序等级变量：<br>（1）.出院；（2）.住院治疗，不需要补充氧气；（3）.住院，需要补充氧气

	（但不需要NIV / HFNC）；（4）.重症监护病房/住院，需要NIV / HFNC治疗；（5）.重症监护病房，要求ECMO和/或IMV；（6）.死亡。（缩写注释： NIV，无创机械通气； HFNC，高流量鼻导管；IMV，有创机械通气。）<br><br>2.次要疗效指标<br><br>（1） 核酸变为阴性的时间， Co201VID-2019 的标记（基线，D1，D4，D7，D14，D28 或出院日）<br>（2） 胸部 CT 显示病变区域的大小（D1，D7，D14，D28 或出院日）<br>（3） 死亡率（D28 天内）<br>（4） 免疫指标变化：（基线，D1，D4，D7，D14，D28 或出院日和随访期 D90±3）<br>✓ 收集外周抗凝血，检测淋巴细胞亚群变化：T淋巴细胞（CD3、CD4、CD8）、B淋巴细胞（CD19）、NK细胞（CD16、CD56）；<br>✓ 收集外周血血清，检测各种免疫球蛋白：IgA、IgG、IgM、总IgE；<br>✓ 收集外周血血清，探索细胞因子的变化规律，Th1类细胞因子（IL-1β、IL- 2、TNF-a、ITN-γ），Th2类细胞因子（IL- 4、IL- 6、IL- 10）。<br><br>（5）C 反应蛋白（基线，D1，D4，D7，D14，D28 或出院日和随访期 D90±3）<br>（6）丙氨酸氨基转移酶和肌酸激酶水平（基线，D1，D4，D7，D14，D28 或出院日和随访期 D90±3）<br>（7）其他一般临床检测指标<br>血氧饱和度、呼吸频率、咳嗽程度测量值、体温。在筛选期、住院期间每日进行（另增加D1、D4、D7给药前30min、给药结束后2h±30min、24h±30min）。<br><br>**咳嗽症状积分表**<br><br>| 分值 | 日间咳嗽症状积分 | 夜间咳嗽症状积分 | |---|---|---| | 0 | 无咳嗽 | 无咳嗽 | | 1 | 偶有短暂咳嗽 | 入睡时短暂咳嗽或又有咳嗽 | | 2 | 频繁咳嗽，轻度影响日常活动 | 因咳嗽轻度影响夜间睡眠 |

	3　频繁咳嗽，严重影响日常活动　因咳嗽严重影响夜间睡眠
统计方法	本研究采用区组随机化方法，由统计人员使用SAS 9.4软件产生随机分组编码，受试对象按照该编码，以1:1的比例被分配至：常规治疗+安慰剂组（对照组）和常规治疗+hDPSC细胞治疗组（试验组）。统计人员不参与筛选、纳入受试者和后续的临床研究。研究过程中对受试者、观察评估人员和统计人员设盲。<br>统计分析数据集包括全分析数据集（FAS）、符合方案数据集（PPS）、安全性数据集（SS）。<br>分析内容包括受试者分布描述、基线指标的均衡性分析、疗效分析、安全性分析。<br>计量资料的统计描述采用均数、标准差、中位数、四分位数、最小值和最大值，组间比较采用方差分析或Kruskal-Wallis秩和检验，组内前后比较采用配对t检验或Wilcoxon符号秩检验。<br>计数与等级资料的统计描述采用率或构成比，计数资料的比较采用x2检验或Fisher精确概率法，等级资料的比较采用Kruskal-Wallis秩和检验。
技术支持	本项目研究用的人牙髓间充质干细胞原液由北京三有利和泽生物科技有限公司具有符合 GMP 生产条件的细胞制备车间提供；在湖北优牙生物科技有限公司完成复苏细胞，用氯化钠注射液洗涤细胞后重悬于 30mL 氯化钠注射液中，即为人牙髓间充质干细胞制剂。在武汉大学人民医院检验科进行放行检验。研究用人牙髓间充质干细胞的分离、培养、鉴定、质控等制备流程均在相应的标准操作规程（SOP）指导下进行，所有细胞均通过严格的企业自检，符合临床级干细胞的质量标准，对于受试者以及研究者均有安全性保证。

（一）研究题目

异体人牙髓间充质干细胞治疗 COVID-19 重症肺炎的安全性和有效性研究 。

（二）研究目的

评价应用人牙髓间充质干细胞（hDPSC）治疗新型冠状病毒所致重症肺炎的安全性和有效性；为探索应用人牙髓间充质干细胞（hDPSC）治疗新型冠状病毒所致重症肺炎，降低新型冠状病毒肺炎死亡率，改善临床预后提供新的治疗方案。

（三）立题依据

新型冠状病毒感染的肺炎是由新型冠状病毒 COVID-19 感染所导致的以发热，咳嗽、呼吸急促和呼吸困难为主要特征的感染性肺炎。患者 CT 显示早期呈现单发或多发小斑片影及间质改变，以肺外带明显，进而发展为双肺多发磨玻璃影，浸润影，严重者可出现肺实变。重型病例多在一周会出现呼吸困难，指氧饱和度下降明显（≤93%），动脉血氧分压（PaO2）/吸氧浓度（FiO2）≤300，危重者快速进展为急性呼吸窘迫综合征，出现休克，合并其他器官功能衰竭。

间充质干细胞（mesenchymal stem cells，MSCs）在体内外可以诱导分化为多种细胞，可以改善多种损伤组织的结构与功能。MSCs 在多种肺脏疾病损伤中都体现出了其治疗潜能[1]。MSCs 用于治疗肺损伤的理论基础包括：（1）无移植排斥反应；（2）移植的 MSCs 可以归巢至受损伤的肺脏，同时在活性因子的作用下，修复损伤的肺泡上皮细胞，改善肺脏局部微环境；（3）调节炎性反应：MSCs 通过其免疫抑制功能（抑制免疫细胞如树突状细胞、T 淋巴细胞、NK 细胞等的激活）和改变细胞因子在疾病炎性过程中的表达谱来发挥其抗炎作用[2]；（4）抗凋亡[3]；（5）通过分泌血管生长因子促血管生成[2,4]；（6）减轻纤维化[5]。

本项目前期研发了人牙髓间充质干细胞制剂，开展了系统的 GLP 临床前安全性评价，结果为安全。同时，项目组利用多种动物模型开展了间充质干细胞治疗肺损伤的实验研究，结果显示间充质干细胞对肺损伤的治疗具有减轻肺脏局部炎症，改善纤维化的作用。不同来源间充质干细胞比较研究显示，hDPSC 较人脐带间充质干细胞具有更强的免疫调节作用，对博来霉素诱导的肺纤维化大鼠模型的治疗研究显示具有相近疗效，个别指标优于脐带间充质干细胞。此外，前期开展的预探索临床研究“人脐带间充质干细胞治疗急性百草枯中毒致肺损伤的临床观察”，可见间

充质干细胞具有积极的肺损伤修复效果。因此，可以预期 hDPSC 在新型冠状病毒感染的肺损伤中有望起到有效的治疗作用。

目前，国内外已经开展了多项 MSCs 治疗肺部疾病的临床试验。2013 年，美国 UCLA 的团队发表了他们利用异基因来源的 MSCs（即 Osiris 公司的 Prochymal 产品）治疗慢性肺阻的效果（NCT00683722），共入组 62 名中度到重度慢性肺阻病人，随机分成接受静脉回输 MSCs 的治疗组和安慰剂组，随后接受为期 2 年的回访。治疗组没有出现回输毒性、死亡或者严重的副作用事件。随访结果显示 MSCs 治疗显著降低 COPD 病人血液中的 C 反应蛋白，显著改善病人系统性炎症的病症[6]。2015 年，美国开展 MSCs 治疗呼吸窘迫综合征的临床研究（NCT01775774)共入组 9 例病例，随机平均分为 3 组，分别给予骨髓来源 MSCs 低、中、高剂量静脉输注。随访 1 年，观察不同组别不良反应事件比例。研究结果显示 3 组均没有发现与移植 MSCs 的严重不良反应。在 MSCs 移植过程中心率、氧饱和度、肺动脉压力改变 3 组无差异[7]。2018 年，第三军医大学西南医院报道了利用脐带来源 MSCs 治疗放射性肺纤维化（radiation-induced pulmonary fibrosis，RPF）的观察结果。入组病人 8 例，通过支气管镜对肺纤维化病灶部位灌洗并单次注入 MSCs。8 位患者均能够耐受 MSCs 治疗，随访观察无严重不良反应发生。6 例患者自述气促、咳嗽等症状有所好转，CT 肺密度下降，说明 MSCs 治疗安全，耐受性好；能够减轻患者的临床症状，降低肺纤维化密度[8]。这些研究显示了应用 MSC 治疗肺脏病变具有非常很好的安全性和疗效。本次新型冠状病毒肺炎疫情发生以来，已有北京、浙江、湖北等团队开展了干细胞用于新型冠状病毒肺炎的治疗研究。国家卫健委也正在积极组织推进干细胞在新型冠状病毒肺炎重症治疗方面的临床疗效研究探索。

参考文献

[1] Lee SH, Jang AS, Kim YE, Cha JY, Kim TH, Jung S, Park SK, Lee YK, Won JH, Kim YH, Park CS. Modulation of cytokine and nitric oxide by mesenchymal stem cell transfer in lung injury/fibrosis. Respir Res. 2010; 11(1):16.

[2] Mei SH J, McCarter SD, Deng YP, Parker CH, Liles WC,Stewart DJ. Prevention of LPS-induced acute lung injury in mice by mesenchymal stem cells overexpressing angiopoietin 1. Plos Med. 2007; 4(9): 1525-37.

[3] Caplan A. Why are MSCs therapeutic? New data: new insight. J Pathol. 2009;217:318-24.

[4] Kursova LV, Konoplyannikov AG, Pasov VV, Ivanova IN, Poluektova MV, Konoplyannikova OA. Possibilities for the use of autologous mesenchymal stem cells in the therapy of radiation-induced lung injuries. Bull Exp Biol Med. 2009;147(4):542-6.

[5] Jun Guo, Guo-sheng Lin, Cui-yu Bao, Zhi-min Hu, Ming-yan Hu. Anti-inflammation role for mesenchymal stem cells transplantation in myocardial infarction. Inflammation. 2007; 30: 97-104.

[6] Weiss DJ，Casaburi R，Flannery RA，et a1．A PlaceboControlled，randomized trial of mesenchymal stem cells in COPD[J1．Chest，2013，143(6)：1590—1598．

[7] Wilson JG，Liu KD，Zhuo H，et a1．Mesenchymal stem (stromal)cells for treatment of ARDS：a phase 1 clinical trial [J]．Lancet Respir Med，2015，3(1)：24—32.

[8] Jiang X，Jiang X，Qu C，et al Intravenous delivery of adipose-derived mesenchymal stromal cells attenuates acute radiationinduced lung injury in rats E[J]. Cytotherapy, 2015, 17(5)：560—570．

（四）预期效果

在控制病毒致肺部感染常规治疗基础上，联合运用人牙髓间充质干细胞治疗，能够提高新型冠状病毒感染致肺损伤修复的临床疗效。

1．临床指标检查

与基线相比，临床指标改善时间TTCI六类有序等级变量逐步下降，至14天下降两个级别或以上。

2．影像学检查

与基线相比，患者胸部 CT 影像病灶大小和 CT 值随着治疗时间的延长逐渐好转，至 D28 可吸收≥70%。

3. **实验室检查**

治疗组连续两次呼吸道 COVID-19 病原核酸检测阴性（采样时间间隔 24 小时）。

（五）研究设计

1．本研究为单中心、随机、对照研究，

临床诊断为新型冠状病毒所致重症肺炎的受试者签署知情同意书并符合纳入标准且不符合排除标准后，所有受试者均接受新冠肺炎常规治疗，并于常规疗同时接受安慰剂或 hDPSC 细胞输注治疗。所有受试者接受研究治疗后观察至 D28 或出院以临床指标改善时间（TTCI）作为主要评价指标。持续随访收集受试者临床安全和有效性观察指标直至结束研究。

2．本研究设 2 个组，按照 1:1 比例随机入组。

2.1 分组设计：

本研究设 2 个组，分别为常规治疗+安慰剂组（对照组），常规治疗+hDPSC 细胞治疗组（试验组），每组 10 例，共入组 20 例受试者。

常规治疗+安慰剂组：该组受试者接受新冠肺炎常规治疗：在对症治疗的基础上，积极防治并发症，治疗基础疾病，预防继发感染，及时进行器官功能支持；呼吸支持；循环支持，同时，分别于 D1、D4、D7 接受 30mL 安慰剂静脉输注治疗，输注采用一次性输血器进行输注。

常规治疗+hDPSC 细胞治疗组：该组受试者在接受常规治疗（同常规治疗+安慰剂组）同时分别于 D1、D4、D7 接受 hDPSC 细胞静脉输注治疗，输注采用一次性输血器进行输注，细胞输注剂量为 3.0×10^7 hDPSC 细胞/人。输注周期为 1 周，每次间隔 2 天。

为控制细胞治疗试验风险，常规治疗+hDPSC 细胞治疗组采取分步实施，首例受试者给药疗程结束（D1-D7）且研究者根据安全性评价指标判断为给药安全时，再进行本组后续受试者的入组治疗；如出现 1 例受试者发生很可能或肯定与试验药物相关的严重不良事件，则终止本试验。

临床诊断为新冠肺炎的受试者签署知情同意书并符合纳入标准且不符合排除标准后，研究者根据统计人员提供的随机分组编码，将受试者随机分配入组。

2.2 细胞治疗剂量设计：根据临床前研究结果和 ClinicalTrials.gov 注册项目“间充质干细胞治疗 2019 新型冠状病毒感染的肺炎患者的安全性和有效性”的给药设计（每例受试者每疗程给予 $1.5\text{-}3.0\times10^6$ 细胞/ kg），本项目每例受试者每次输注细胞剂量为：约 3.0×10^7 细胞/人，每 3 次细胞输注为 1 个治疗疗程（按每例受试者每疗程 1.5×10^6 细胞/ kg 计算）。

2.3 随机方法：

本研究采用完全随机化方法，由统计人员使用 SAS 9.4 软件产生随机分组编码，受试对象按照该编码，以 1:1 的比例被分配至：常规治疗+安慰剂组，常规治疗+hDPSC 细胞治疗组。

3.盲法设置：本研究采取单盲，对受试者和临床观察评价人员、统计人员设盲。

4.研究步骤：

（1）筛选期（D-7-0）：

【签署知情同意书】：研究者充分告知受试者关于本研究的详细信息，新冠肺炎的治疗选择，可能的风险与获益后，获得受试者书面、自愿签署的知情同意书后，受试者纳入受试者筛选。

【纳入/排除标准】：筛选符合入组标准且不符合排除标准的受试者。

【人口学资料】：采集并记录受试者的人口统计学资料，包括性别、出生日期、民族、身高、体重。

【病史资料】：采集并记录受试者现病史、过敏史、家族史、既往史（如吸烟史、糖尿病史、其他手术史等）。

【生命体征】：心率、血压（收缩压、舒张压）。

【实验室检查】：

- **血常规**：白细胞、红细胞、血红蛋白、红细胞压积、平均红细胞体积、平均血红蛋白量、平均血红蛋白浓度、血小板计数、血小板平均体积、血小板压积、中性粒细胞、淋巴细胞、单核细胞、嗜酸细胞、嗜碱细胞、中性粒细胞绝对值、淋巴细胞绝对值、单核细胞绝对值、嗜酸细胞绝对值、嗜碱细胞绝对值、红细胞分布宽度SD、红细胞分布宽度CV、大血小板比率、血小板分布宽度；
- **肝肾功能**：丙氨酸氨基转移酶、天门冬氨酸氨基转移酶、总蛋白、白蛋白、球蛋白、白球比、总胆红素、直接胆红素、间接胆红素、碱性磷酸酶、肌酐、γ-谷氨酰基转移酶、乳酸脱氢酶、肌酸激酶、尿素、尿酸、总二氧化碳、葡萄糖；
- **炎症指标**：超敏C反应蛋白、血清淀粉样蛋白（SAA）；
- **传染病检测**：乙肝（HBsAg、HBsAb、HBeAg、HBeAb、HBcAb）、丙肝（Anti-HCV）、艾滋病（HIVcombin）、梅毒（Anti-TP）、巨细胞病毒CMV-IgM、巨细胞病毒CMV-IgG；
- 免疫学检测：
  - ✓ 收集外周抗凝血，检测淋巴细胞亚群水平：T淋巴细胞（CD3、CD4、CD8）、B淋巴细胞（CD19）、NK细胞（CD16、CD56）；
  - ✓ 收集外周血血清，检测各种免疫球蛋白水平：IgA、IgG、IgM、总IgE；
  - ✓ 收集外周血血清，检测细胞因子水平，Th1类细胞因子（IL-1β、IL- 2、TNF-a、ITN-γ），Th2类细胞因子（IL- 4、IL- 6、IL- 10）。
- **妊娠试验**：血β-HCG，绝经期前的女性受试者；
- **尿常规**：PH值、比重、蛋白质、酮体、胆红素、尿胆素原、亚硝酸盐、白细胞、红细胞、尿糖、尿沉渣镜检；
- **便常规**：颜色、性状、白细胞、红细胞、脂肪球、虫卵、真菌、潜血（出院后不检测）。

【临床检测指标】：血氧饱和度、呼吸频率、咳嗽程度测量值、体温。

【影像学检查】胸部 CT。早期呈现单发或多发小斑片影及间质改变，以肺外带明显，进而发展为双肺多发磨玻璃影，浸润影，严重者可出现肺实变。

【新冠病毒（COVID-19）检测】：采用实时定量 PCR 试剂盒进行检查。

【随机分组】：在 D-2 天前，对符合入组标准的受试者，研究者根据统计人员提供的随机分组编码，给予受试者随机分配号，确定受试者组别。

（2）治疗与观察期（D1-D28 或出院日）：

【纳入/排除标准】：核对受试者是否符合入组标准

【生命体征】：心率、血压（收缩压、舒张压）。住院期间每日进行检查（另在 D1、D4 和 D7 细胞输注治疗前 30min、细胞输注治疗结束后 2h±30min、24h±30min 分别增加生命体征检查）。

【六类有序等级变量】：住院期间每日进行检查。

①.出院；②住院治疗，不需要补充氧气；③住院，需要补充氧气（但不需要NIV / HFNC）；④重症监护病房/住院，需要NIV / HFNC治疗；⑤重症监护病房，要求ECMO和/或IMV；⑥死亡。（ NIV，无创机械通气； HFNC，高流量鼻导管；IMV，有创机械通气。）

【临床检测指标】：血氧饱和度、呼吸频率、咳嗽程度测量值、体温。在住院期间每日进行检查（另增加D1、D4、D7给药前30min、给药结束后2h±30min、24h±30min检查）。

咳嗽症状积分表：

分值	日间咳嗽症状积分	夜间咳嗽症状积分
0	无咳嗽	无咳嗽
1	偶有短暂咳嗽	入睡时短暂咳嗽或又有咳嗽
2	频繁咳嗽，轻度影响日常活动	因咳嗽轻度影响夜间睡眠
3	频繁咳嗽，严重影响日常活动	因咳嗽严重影响夜间睡眠

【影像学检查】胸部 CT，在 D1、D7、D14、D28 或出院日进行。

【新冠病毒（COVID-19）检测】：D1，D4，D7，D14，D28 采用实时定量 PCR 试剂盒检测核酸，若一次阴性则间隔 24 小时再次采样，若连续 2 次检查结果阴性则结束检测。

【实验室检查】：在 D1、D4、D7 给药前 30min、给药结束后 2h±30min、

24h±30min、D14、D28 天或出院日进行。包括与筛选期相同的血常规、肝肾功检查、炎症指标检查、免疫学检查、尿便常规检测项目。

【研究治疗】:

- **常规治疗组+安慰剂组：**仅接受常规治疗。在对症治疗的基础上，积极防治并发症，治疗基础疾病，预防继发感染，及时进行器官功能支持；呼吸支持；循环支持等；同时分别于D1、D4、D7接受30mL安慰剂静脉输注治疗，输注采用一次性输血器进行输注，研究者记录受试者常规治疗的内容和起止时间以及安慰剂静脉输注治疗的起止时间。
- **常规治疗+hDPSC细胞治疗组：**该组受试者在接受常规治疗（同常规治疗组）的同时，分别于D1、D4、D7接受hDPSC细胞静脉输注，输注采用一次性输血器进行输注，细胞输注剂量为3.0×10^{7} hDPSC细胞/人，细胞的输注周期为1周，每次间隔2天。研究者记录受试者常规治疗的内容和起止时间以及细胞制剂静脉输注治疗的起止时间。

研究给药时，研究护士核对患者姓名、制剂标签信息，采血袋轻轻摇晃4-5次确认肉眼无细胞聚集后，采用一次性输血器输注。操作过程中注意无菌原则和输血器具的密封性。输血器的接头一般与7-8#头皮针或22G静脉留置针连接。静脉通路一般选择前臂或肘部粗、直、弹性好的外周静脉，如贵要静脉、肘正中静脉、头静脉等，外周静脉能够保证干细胞输注速度的要求。输注前生理盐水冲管10min后，输注细胞，最初5-10min内缓慢输注，滴速应调控在15滴/min左右，防止细胞聚集堵塞微血管，如无不适反应,增加输注滴速，30ml干细胞制剂在20-30min内输完。输完后可用一定量的生理盐水冲洗管路输注，以尽可能将残留的干细胞输注。输注期间和输注后研究医师应密切关注患者的情况，防止急性输液反应的发生，输注期间和输注后常规心电监测，观察患者输注期的脉搏、血压和血氧饱和度的变化，同时测量患者输注前后的体温变化。

【不良事件记录】：治疗期间记录不良反应。

【合并用药/治疗的记录】

患者体温恢复正常3天以上，呼吸道症状明显好转，肺部影像学明显吸收，连续两次呼吸道病原核酸检测阴性（采样时间间隔24小时），可结束治疗出院。

（3）随访期（D90±3）

【生命体征】：呼吸、心率、血压（收缩压、舒张压）、体温（腋下）。

【实验室检查】：同筛选期，包括血常规、肝肾功检查、炎症指标检查、免疫学检查、尿常规、传染病检查、血妊娠检查。

【影像学检查】：胸部 CT 检查。

【新冠病毒（COVID-19）检测】：采用实时定量 PCR 试剂盒进行检查。

【不良事件记录】

【合并用药/治疗的记录】

（六）研究实施条件

1.研究人员条件：

本研究团队参与的研究者均具有多年丰富的临床和科研经验，且都完成药物临床试验质量管理规范（GCP）培训并获得相应证书。

1）叶青松：医学科学博士，主任医师，教授，研究员，博士生导师，中组部青年千人计划入选者，爱丁堡皇家外科学院院士、英国皇家医学会会士（遴选）、国际牙医师学院院士、国际循证医学 Cochrane 协作网成员。现任武汉大学人民医院再生医学中心主任、人民医院临床研究（干细胞研究）学术委员会副主任委员、东院重症医学科干细胞治疗专家。曾任澳大利亚昆士兰大学牙学院终身教授、科研副院长、医院伦理委员会主任。从事循证医学和临床科研设计工作 18 年，作为国际循证医学 Cochrane 协作网成员以及中国循证医学中心兼职编辑多次参加临床科研设计方法与质量评估的会议和作报告。在临床科研项目设计、质量控制和数据分析等方面有较深的造诣。从事牙髓干间充质细胞研究近 10 年，对牙髓间充质干细胞的提取、培养、分化以及临床应用进行了深入地研究，受邀担任《Stem Cells International》专刊主编、《Journal of Investigative & Clinical Dentistry》副主编。发表论文 87 篇、出版、参编专著 3 部。目前主持的与干细胞相关的课题研究三项：一种仿病毒结构的无机纳米基因转运系统在干细胞三维组织工程中的应用研究（国自然目上项目）；可注射水凝胶携载纳米银微粒和牙髓间充质干细胞在慢性输卵管炎治疗中的应用研究；牙髓间充质干细胞在视神经损伤中的应用研究。

2）周晨亮：医学博士，东院重症医学科主任，副主任医师，发表论文 8 篇。多次参与药物临床试验培训班，拥有药物临床试验质量管理规范证书。在此次新冠肺炎

的治疗中，其领导的科室（隔离病房）是重型/危重型患者的指定救治点。周主任在一线救治病人的过程中积累了大量的临床经验，对新冠肺炎患者的病程发展、治疗方法和转归有深刻的认识，可以有效地领导本研究方案在临床的贯彻和执行。参与科研项目两项：湖北省自然科学基金和湖北省卫生和计划生育委员会面上项目。还参与了两项新冠肺炎的临床试验：雾化吸入喜炎平注射液治疗新型冠状病毒肺炎（COVID-9）的临床研究 注册号：ChiCTR2000029756；羟氯喹对新型冠状病毒肺炎（COVID-9）的治疗疗效研究 注册号：ChiCTR2000029573；有着丰富的课题研发和临床试验经验。

3）张旃：医学博士，呼吸与危重症医学二科副主任医师，科党支部书记。中国救援协会重症医学分会常务理事、中国救援协会重症医学分会呼吸治疗组副组长、中国医药教育协会呼吸病运动康复分会理事、湖北省医学会临床药学分会第二届委员会委员。发表论文 10 余篇，获得自然基金项目 5 项，参与了两项新冠肺炎的药物临床试验：雾化吸入喜炎平注射液治疗新型冠状病毒肺炎（COVID-9）的临床研究 注册号：ChiCTR2000029756；羟氯喹对新型冠状病毒肺炎（COVID-9）的治疗疗效研究 注册号：ChiCTR2000029573

2.研究场所、仪器条件：

本研究在武汉大学人民医院东院区进行受试者招募，能够保证足够数量的符合纳入标准且不符合排除标准的受试者入组。能够提供独立的空间进行受试者的入组前谈话和治疗。

本研究所用干细胞制剂的放行检验由医院检验科完成。制剂放行检验项目为：细胞数、细胞活率，需要用到的仪器为倒置显微镜，试剂为氯化钠注射液、台盼蓝染液。医院检验科完成实验室满足干细胞制剂放行检验的条件。

干细胞制剂在研究机构由负责干细胞制剂管理与分发的研究者接收后，直到受试者接受干细胞制剂输注治疗的时间段内，储存在符合条件且可记录温度的冰箱中。保存条件为：4℃。保存时间为：6 小时。干细胞输注治疗完成后，打印干细胞制剂运输与储存过程温度记录单并存档。

本研究安全性评估指标中的实验室检查由医院检验科完成，本研究有效性评估指标中的影像学检测均采用 CT 检查，由放射科完成。

（七）受试者纳入、排除标准和分配入组方法

1. 纳入标准：受试者必须满足以下所有标准，方可纳入本研究：

（1） 年龄 18-65 周岁，性别不限；

（2） 自愿参加本临床研究并签署《知情同意书》；

（3） 诊断为重型新冠肺炎：呼吸窘迫，RR≥30 次/分；静息状态下，指氧饱和度≤93%；动脉血氧分压/吸氧浓度≤300mmHg；新冠病毒（COVID-19）核酸检测为阳性。

（4） 胸部影像检查证实肺受累。

2. 排除标准：符合下列任意一项的受试者，将被排除在此项研究之外：

（1） 在筛选评估之前的30天内接受任何针对COVID-19的临床试验药物治疗者；

（2） 严重肝病（例如Child Pugh分数≥C或AST>上限的5倍）；

（3） 已知严重肾功能不全者（估计肾小球滤过率≤30mL/min/1.73 m2）或接受连续性肾脏替代治疗，血液透析，腹膜透析的患者；

（4） HIV，乙肝，结核，流感病毒，腺病毒和其他呼吸道感染病毒的共同感染；

（5） 研究筛选前一个月之内没有保护措施的性生活的女性患者；

（6） 妊娠、哺乳期妇女或应用雌性激素避孕的女性；

（7） 本人或配偶计划在研究期间和研究结束后 6个月内妊娠的患者；

（8） 研究者认为其他不适宜参加的情况。

3.退出/脱落标准：

（1） 受试者在研究过程中发生了严重不良事件，研究者认为不能再继续进行研究；

（2） 受试者依从性差，不能按时完成随访；

（3） 受试者不愿继续进行临床研究，向研究者提出退出；

（4） 失访。

4.分配入组方法：本研究采用完全随机化方法，由统计人员使用 SAS 9.4 软件产生随机分组编码，受试对象按照该编码，以 1:1 的比例被分配至常规治疗对照组和

常规治疗+hDPSC 细胞治疗组。统计人员不参与筛选、纳入受试者和后续的临床研究。

（八）所需的病例数

鉴于本项目为探索性临床研究，样本含量估算以主要疗效指标变化的现有临床经验为基础，hDPSC 细胞治疗试验组、和常规治疗对照组每组需要 8 例研究对象，考虑 20%的脱落率，每组例数为 10 例，总例数为 20 例。

（九）受试者管理

1.**受试者的招募方式：**本研究临床诊疗过程中进行受试者的招募，由经主要研究者授权的具备资质的临床医师进行。

2.**知情同意过程：**负责招募的研究者在独立的治疗室对有意向参加本研究的患者或其法定代理人进行项目介绍，详细解释本研究的要求、给药过程和步骤，以及参加本研究后可能带来的风险和不适，同时告知患者或其法定代理人参加本研究是自愿选择的，患者有权选择参加本研究或者选择其他治疗方案，可以和亲属、朋友联系讨论是否参加本研究，也可在任意时间退出。研究者在进行患者入组前谈话的过程中，应该回答患者或其法定代理人有关本研究的所有问题直到患者或其法定代理人充分了解本项目，并保证患者或其法定代理人有足够的时间考虑是否参加本研究。

患者或其法定代理人同意参加本研究后，正式成为本研究的受试者，与研究者共同签署经武汉大学人民医院临床研究伦理委员会批准的最新版知情同意书（注明版本号和版本日期），双方均签署姓名并注明日期。同时研究者应告知患者或其法定代理人，在研究过程中，如发现涉及干细胞制剂新的重要信息则研究组必须将知情同意书作书面修改，并经武汉大学人民医院临床研究伦理委员会批准后，将再次取得患者或其法定代理人的知情同意。

3.**筛选编号的分配：**研究者根据受试者的筛选顺序，给与受试者筛选编号。

4.**核对入排标准：**研究者筛选符合纳入标准且不符合排除标准的受试者，对入组的受试者进行全面评估，核对受试者是否满足所有的筛选入组条件。

5.**治疗/随机分组编号的分配：**研究者在筛选期确认受试者满足所有的入组条件后，根据统计人员提供的随机分组编码，给予受试者随机分配号，确定受试者组

别，并在基线/治疗期再次确认受试者满足所有的入组条件，告知受试者最终入组信息。

6.研究依从性管理：受试者取得随机分配号并分配给本研究项目的研究者后，由研究者负责受试者的跟踪随访。研究者应认真执行本临床研究方案和知情同意书，使受试者充分理解研究要求，配合研究，并定期电话提醒受试者随访时间。

7.报酬与补偿：受试者参加本研究将获得 300 元的费用作为参加本研究进行出院后随访检查的交通和误工补偿，由研究者负责在出院后随访访视结束以后以现金方式发放给受试者。

（十）受试者激励与补偿

1.研究费用：受试者因参加本研究进行的与本研究相关的检查、治疗费用由专项项目经费支付，不足部分由研究单位武汉大学人民医院支付；受试者因参加本研究进行的干细胞输注治疗的制剂制备费用、相关数据管理与统计分析费用、受试者保险费用由专项项目经费支付，不足部分由制剂合作单位支付。

2.研究相关损害的治疗与补偿/赔偿：研究相关损害是指完全为实现研究目的而执行研究程序或干预措施造成的损害，受试者应获得相应的补偿/赔偿和/或免费医疗。补偿是指在研究者没有违规责任的合法民事行为但是给对方造成一定损失的情况下进行的，而赔偿是在研究者的违规责任行为造成对方人身或财产损害的情况下进行的。

如果受试者在研究期间发生与本研究相关的不良事件，由研究者对其进行相应的免费医疗救治，并同时告知制剂制备机构，必要时由医疗事故鉴定委员会对不良反应的性质和程度进行鉴定和评估。一旦发生研究相关损害，由北京三有利和泽生物科技有限公司购买的受试者保险进行相应的补偿/赔偿。

（十一）受试者隐私与保密

隐私：本研究为涉及人的生物医学研究，在研究过程中需要采集受试者的私人信息，包括：与个人身份相关的信息，如姓名、性别、出生日期、民族、身高、体重、电话号码、证件（身份证、社会保障卡）号、门诊号、书写的签名等；个人健康相关的信息，如个人的医疗记录，如现病史、过敏史、家族史、既往史等。研究者在诊疗过程中收集以上个人信息是在履行医生这一特殊职业的职责，目的是为正

确诊断和有效治疗患者的疾病。因此，在本研究中，研究者收集个人信息并不必然构成侵害受试者隐私权的行为。但是，在研究中收集的个人信息的使用是有特殊限制的，超出适用范围，须经受试者同意。

1.研究样本、资料、数据：

在研究期间，受试者的研究样本（如静脉血样）、研究资料（所有纸质记录和电子记录的文件）、以及所有的研究数据在任何时候都将被严格保密。常规的保密措施有：①培训研究人员保密的重要性；②给受试者的样本、研究资料以及所有的研究数据去标识化：给予每位受试者筛选编号，每位受试者的原始病历和病例报告表均采用筛选编号与姓名缩写记录数据与信息；③将本研究所有的研究文件放在专用的柜子里上锁，指定专门的研究者负责；④研究文件与数据锁定后，限制文件与数据的查看权限。这些信息仅限于在必要时提供给本项目的研究者、伦理委员会以及国家药品监督管理部门。

在研究结束后，研究机构应按照国家有关法规的规定保存所有本临床研究项目的资料，包括本研究项目的临床研究方案、知情同意书、原始病历、病例报告表、研究者手册、学术委员会批件、伦理委员会批准件、研究协议、干细胞制剂分发的详细记录、不良事件及严重不良事件记录等。所有记录保存期限按国家药政管理部门的要求执行。

2.研究结果发表/公开：研究结束后，项目组会发表研究论文或公开其他形式的研究成果。在发表的研究论文或公开的研究成果里，受试者的个人信息将被隐藏，受试者的姓名或其他任何可识别出受试者身份的内容都将不会出现，也不会在将来的任何时候泄露。

（十二）干细胞制剂的使用方式、剂量、时间及疗程

1.研究制剂的制备：

- 干细胞制剂：该干细胞制剂原液由北京三有利和泽生物科技有限公司在具有 GMP 生产条件的细胞生产车间内制备。使用前在湖北优牙生物科技有限公司具有 GMP 生产条件的细胞生产车间内复苏细胞，用氯化钠注射液洗涤细胞后重悬于 30mL 氯化钠注射液中即为 hDPSC 细胞制剂，制剂规格：约为 3×10^{7} hDP-MSC 细胞/30 mL /袋。

- 安慰剂制剂：由北京三有利和泽生物科技有限公司在具有 GMP 生产条件的细胞生产车间内制备，用 30mL 氯化钠注射液为安慰剂制剂，制剂规格：30 mL /袋。

2.研究制剂治疗剂量与治疗方式：

- hDPSC 细胞治疗：分别于 D1、D4、D7 给予受试者 hDPSC 细胞静脉输注治疗，输注采用一次性输血器进行输注，每次治疗剂量为 3.0×10^7 hDPSC 细胞，细胞悬浮于 30mL 生理盐水静脉滴注。

- 安慰剂治疗：分别于 D1、D4、D7 给予受试者安慰剂静脉输注，输注采用一次性输血器进行输注，每次 30mL 生理盐水静脉滴注治疗。

研究给药时，研究护士核对患者姓名、制剂标签信息，采血袋轻轻摇晃 4-5 次确认肉眼无细胞聚集后，采用一次性输血器输注。操作过程中注意无菌原则和输血器具的密封性。输血器的接头一般与 7-8#头皮针或 22G 静脉留置针连接。静脉通路一般选择前臂或肘部粗、直、弹性好的外周静脉，如贵要静脉、肘正中静脉、头静脉等，外周静脉能够保证干细胞输注速度的要求。输注前生理盐水冲管 10min 后，输注细胞，最初 5-10min 内缓慢输注，滴速应调控在 15 滴/min 左右，防止细胞聚集堵塞微血管，如无不适反应,增加输注滴速，30ml 干细胞制剂在 20-30min 内输完。输完后可用一定量的生理盐水冲洗管路输注，以尽可能将残留的干细胞输注。输注期间和输注后研究医师应密切关注患者的情况，防止急性输液反应的发生，输注期间和输注后常规心电监测，观察患者输注期的脉搏、血压和血氧饱和度的变化，同时测量患者输注前后的体温变化。

3.研究制剂治疗疗程：本研究 3 次静脉输注给药为一个疗程，时间为 1 周，每次治疗间隔 2 天。

（十三）研究制剂管理

1.干细胞制剂出库：研究者应用干细胞制剂需提前 2 天向湖北优牙生物科技有限公司申请，并明确数量。制剂制备机构完成制备后，须立即进行质量检验。干细胞制剂连同质检报告一同出库，制剂的检验项目包括：外观、细胞数、细胞活率、无菌试验、细菌内毒素含量、牛血清白蛋白残留、二甲基亚砜等。其中无菌试验、牛血清白蛋白残留、二甲基亚枫这三项检验结果可以滞后。（备注：制剂制备

机构每批干细胞制剂多生产 1 支，用于放行检验和留样追溯。）

2. 干细胞制剂接收：制剂制备机构人员负责保证制剂在4℃的条件下运输并记录温度。制剂到达研究机构时，制剂制备机构人员与负责干细胞制剂管理的研究者进行交接并记录，同时核对如下信息：

- ■ 干细胞制剂包装完整、无破损，标签清晰可辨。
- ■ 干细胞制剂外观：摇匀后为淡乳白色液体，不含肉眼可见不溶物。
- ■ 干细胞制剂标签信息完整：标明为临床研究专用，注明研究方案编号、制剂名、规格、制剂批号、储存条件、使用方法、生产日期、有效期限、制剂制备机构等内容。
- ■ 质检报告信息：制剂名、规格、制剂批号与干细胞制剂标签一致，各检验项目均合格。
- ■ 温度监控记录：查看温度记录仪的温度是否符合制剂稳定性要求，记录最高值、最低值、当前值，并在干细胞制剂局部注射治疗完成后打印制剂运输与储存过程的温度记录单。
- ■ 交接完毕，填写《干细胞制剂接收、回收登记表》。

3.干细胞制剂放行：制剂制备机构每批干细胞制剂多生产 2 支小样（1mL/支），用于机构放行检验和留样追溯。负责干细胞制剂管理与分发的研究者将制剂交与干细胞制剂质控人员；随后干细胞制剂质控人员在医院检验科进行放行检验，检验项目包括：外观、细胞数、细胞活率。由机构质量授权人签署放行检验报告后将放行检验报告存档，同时放行检验合格的干细胞制剂发放研究者临床应用。

4. 安慰剂管理：安慰剂一次性制备、质检放行（无菌、内毒素检测）、常温运输、发放至研究中心药物管理人员，预留 2 袋用于留样追溯。

5. 研究制剂回收：回收制剂包括：注射用后制剂空瓶、未使用剩余制剂以及不合格制剂。制剂制备机构人员与负责干细胞制剂/安慰剂管理与分发的研究者共同核对回收的干细胞制剂标签信息，确认无误后放入运输箱。同时填写《干细胞制剂接收、回收登记表》。

（十四）中止和终止临床研究的标准

1.中止标准：临床研究过程中一旦发现感染、疾病进展，将严格按照事先规定

的预期不良事件处理措施进行处理，并分析该不良反应与干细胞制剂之间的关系，全面评估继续进行本临床研究的风险，如果继续研究会给受试者带来较大或较严重的风险，则中止该研究。

2. 终止标准：临床研究过程中一旦发现以下任一问题，则终止该研究：

（1）研究中发生严重安全性问题（如出现严重不良反应者或出现严重并发症或病情迅速恶化者）；

（2）研究中发现效果较差，甚至无效，不具有临床价值，继续进行下去会延误受试者治疗；

（3）研究中发现临床研究方案制订有重大失误，或方案实施有重大偏差，再继续下去难以评价。

（十五）安全性与疗效评定标准

所有受试者接受研究治疗后均进行安全性、有效性评价，治疗期观察评价至D28 或出院，持续随访收集受试者临床安全和有效性观察指标直至 D90±3 结束研究。

1.安全性评价指标

（1）生命体征：呼吸、心率、血压（收缩压、舒张压）。在筛选期、住院期间每日（另外增加D1、D4、D7输注前30min、输注结束后2h±30min、24h±30min）和D90±3进行。

（2）实验室检查：在筛选期、D1、D4、D7给药前30min、给药结束后2h±30min、24h±30min、住院期间D10、D14和出院后D30±3和D90±3进行。

- 血常规：白细胞、红细胞、血红蛋白、红细胞压积、平均红细胞体积、平均血红蛋白量、平均血红蛋白浓度、血小板计数、血小板平均体积、血小板压积、中性粒细胞、淋巴细胞、单核细胞、嗜酸细胞、嗜碱细胞、中性粒细胞绝对值、淋巴细胞绝对值、单核细胞绝对值、嗜酸细胞绝对值、嗜碱细胞绝对值、红细胞分布宽度SD、红细胞分布宽度CV、大血小板比率、血小板分布宽度；
- 肝肾功能：丙氨酸氨基转移酶、天门冬氨酸氨基转移酶、总蛋白、白蛋白、球蛋白、白球比、总胆红素、直接胆红素、间接胆红素、碱性磷酸酶、肌酐、γ-谷氨酰基转移酶、乳酸脱氢酶、肌酸激酶、尿素、尿酸、总二氧化碳、葡

萄糖；

- 炎症指标：超敏C反应蛋白、血清淀粉样蛋白（SAA）；
- 传染病检测：乙肝（HBsAg、HBsAb、HBeAg、HBeAb、HBcAb）、丙肝（Anti-HCV）、 艾滋病（HIVcombin）、梅毒（Anti-TP）、巨细胞病毒CMV-IgM、巨细胞病毒CMV-IgG；仅在筛选期、随访期D90±3检查。
- 免疫学检测：
  - ✓ 收集外周抗凝血，检测淋巴细胞亚群变化：T淋巴细胞（CD3、CD4、CD8）、B淋巴细胞（CD19）、NK细胞（CD16、CD56）；
  - ✓ 收集外周血血清，检测各种免疫球蛋白变化：IgA、IgG、IgM、总IgE；
  - ✓ 收集外周血血清，探索细胞因子的变化规律，Th1类细胞因子（IL-1β、IL-2、TNF-a、ITN-γ），Th2类细胞因子（IL- 4、IL- 6、IL- 10）。
- 妊娠试验：血β-HCG，绝经期前的女性受试者在筛选期、随访期D90±3检查。
- 尿常规：PH值、比重、蛋白质、酮体、胆红素、尿胆素原、亚硝酸盐、白细胞、红细胞、尿糖、尿沉渣镜检；
- 便常规：颜色、性状、白细胞、红细胞、脂肪球、虫卵、真菌、潜血（出院后不检测）

（3） 不良事件和严重不良事件：研究期间及时记录不良事件和严重不良事件发生情况，不良事件程度按NCI CTCAE v5.0分级标准判定，由研究者判断是否为给药相关不良反应。

2.疗效评价指标

（1）主要疗效指标

临床改善时间（TTCI）：观察自D1-D28天或出院

TTCI的定义是开始研究治疗（阳性药或安慰剂）从入院临床状态的以下六类有序等级变量【（1）出院到（6）死亡】中下降两个等级所经历的时间（天）。六类有序等级变量为：

①.出院；②住院治疗，不需要补充氧气；③住院，需要补充氧气（但不需要NIV / HFNC）；④重症监护病房/住院，需要NIV / HFNC治疗；⑤重症监护病房，要求ECMO和/或IMV；⑥死亡。（ NIV，无创机械通气； HFNC，高流量鼻导管；IMV，有创机械通气。）

（2）次要疗效指标

1) 新冠病毒（COVID-19）检测：核酸变为阴性的时间，COVID-19 的检测（基线，D1，D4，D7，D14，D28 或出院日、D90±3）

2) 胸部 CT 显示病变区域的大小（基线，D4，D7，D14，D28 或出院日），以筛选期影像作为基线，D90±3 的测量值作为评价终点。

3) 死亡率（D28 天内）

4) 免疫指标变化：（基线，D1，D4，D7，D14，D28或出院日和随访期D90±3）

✓ 收集外周抗凝血，检测淋巴细胞亚群变化：T淋巴细胞（CD3、CD4、CD8）、B淋巴细胞（CD19）、NK细胞（CD16、CD56）;

✓ 收集外周血血清，检测各种免疫球蛋白变化：IgA、IgG、IgM、总IgE；

✓ 收集外周血血清，探索细胞因子的变化规律，Th1类细胞因子（IL-1β、IL- 2、TNF-a、ITN-γ），Th2类细胞因子（IL- 4、IL- 6、IL- 10）

5) C 反应蛋白（基线，D1，D4，D7，D14，D28 或出院日和 D90±3）

6) 丙氨酸氨基转移酶和肌酸激酶水平（基线，D1，D4，D7，D14，D28 或出院日和 D90±3）

7) 其他一般临床检测指标：血氧饱和度、呼吸频率、咳嗽程度测量值、体温。在筛选期、住院期间每日进行（另增加D1、D4、D7给药前30min、给药结束后2h±30min、24h±30min）。

咳嗽症状积分表

分值	日间咳嗽症状积分	夜间咳嗽症状积分
0	无咳嗽	无咳嗽
1	偶有短暂咳嗽	入睡时短暂咳嗽或又有咳嗽
2	频繁咳嗽，轻度影响日常活动	因咳嗽轻度影响夜间睡眠
3	频繁咳嗽，严重影响日常活动	因咳嗽严重影响夜间睡眠

（十六）不良事件的记录要求和严重不良事件的报告方法、处理措施

1.安全性数据收集

通过记录、报告和分析基线状况、不良事件、生命体征，实验室检查和肺功能检查评估干细胞制剂的安全性。在整个研究过程中，从受试者签署知情同意开始，

将综合评估受试者出现的任何不良事件。

无法进行安全性评价的受试者可由新入组受试者替代，如未按方案要求完成研究的受试者，包括提前退出、不符合入排标准、未按方案规定完成治疗和访视者。

受试者可以在任何时间无理由退出研究。若受试者依从性差或违背方案，研究者也可以自行决断是否让该受试者退出研究。

2.不良事件（AE）

不良事件（AE）：是指受试者在临床研究过程中出现的所有不良医学事件，可以表现为症状体征、疾病或实验室检查异常，不一定与治疗有因果关系。

注释：在受试者获得参加临床研究知情同意书前已经存在的但在研究过程中严重程度并未发生恶化或者实施进入研究之前即已计划的手术或住院不属于不良事件。

3.不良事件的相关性判断

AE 与研究药物的关系按“肯定有关、很可能有关、可能有关、可能无关、肯定无关”五级判断，前三级列入不良反应发生率计算。

肯定有关：AE 符合研究用药常见的不良反应类型，其出现与研究用药有合理的时间关系，停药后 AE 消失，AE 与合并用药或非药物因素（原发病、并发症、食物、环境等）无关。

很可能有关：AE 符合研究用药常见不良反应类型，与研究用药有合理的时间关系，停药后 AE 明显缓解，AE 与合并用药或非药物因素无关。

可能有关：AE 符合研究用药常见不良反应类型，与研究用药有合理的时间关系，停药后 AE 缓解不明显，不能排除合并用药或非药物因素与 AE 的关系。

可能无关：不良反应与研究用药有合理的时间关系，但不符合研究用药常见不良反应类型，停药后 AE 无缓解，合并用药或非药物因素能解释该 AE。

无关：AE 与研究用药无合理的时间关系，不符合研究用药常见不良反应类型，停药后 AE 无缓解，AE 与合并用药或非药物因素肯定有关。

4.预期不良事件及处理预案

预期不良事件：为受试者参加研究可能面临的不良事件。本研究中可能出现的不良事件及处理措施如下：

（1） 全身不良事件：畏寒、发热、头痛等。输注过程中研究护士持续测量受试者体温，做好记录，若输注过程中出现体温升高、寒战、头痛等

现象，应立即终止输注细胞制剂，评估判断是否感染引起并做相应处理；轻度反应（体温≤37.5℃、轻度寒战、头痛）可予以持续观察，重度反应（体温≥38.5℃、寒战、头痛）给予及时对症治疗。

（2） 疾病进展：在研究期间，受试者如新冠肺炎病情持续进展，由研究者判断干预与否。如采用可能影响研究的其他治疗方法或药物进行干预，或由研究者评估是否退出。

（3） 肺栓塞：在细胞输注过程中，可能会引起肺栓塞，根据临床前研究证实治疗方案的输注剂量一般情况不会引起肺栓塞，但不排除重症肺炎疾病本身出现肺栓塞情况，输注过程会有医师陪伴，密切关注病人情况，无论哪种原因引起的肺栓塞，按照肺栓塞治疗原则及时给予治疗，并由专家组进行评估。

（4） 过敏反应。症状较轻者表现为用药部位红肿，皮肤红斑、荨麻疹等，持续观察可不进行药物干涉或为减轻受试者心理负担给予抗过敏治疗；重度过敏者可能会出现血管神经性水肿、喉痉挛、呼吸困难，更严重者会发生过敏性休克。一旦受试者出现过敏性休克，按照医院《过敏性休克急救预案标准操作》进行救治，详见“备案资料第十三号”。

5.非预期不良事件

非预期不良事件：临床研究过程中发生的同时符合以下 3 条标准的事件：①性质、严重程度和发生率是非预期的；②与参加研究有关或可能有关；③让受试者或他人面临更大风险。新冠肺炎重症患者因疾病进展可能发生的需与非预期不良事件鉴别的不良事件有：严重呕吐、严重腹泻、急性呼衰、急性心衰、急性肝衰、急性肾衰时，一旦发生，研究者首先立即终止干细胞输注，按照医院应急处理流程和相应急救诊疗规范进行急救处理，然后由研究者或专家组判断评估是否与研究治疗有关并作出是否退出或终止研究的决定。

在临床研究中，当多个受试者出现相同的不良事件，而在目前的研究者手册或方案中没有提到其性质、严重程度和频度与研究药物有关，研究者应尽快向制剂制备机构报告这一不良事件。如确诊这一不良事件为非预期药物不良反应，研究者应协助制剂制备机构写出安全性报告交药品监督管理部门和伦理委员会，并通报所有研究者，必要时终止临床研究或对方案及知情同意书进行修正，同时修改研究者手

册，使其包括新的不良反应或已知不良反应的频度和严重程度的变化。

6.严重不良事件（SAE）

临床研究过程中发生需住院治疗、延长住院时间、伤残、影响工作能力、危及生命或死亡、导致先天畸形等事件。

注释：定义中的“危及生命”是指受试者在发生该事件时有死亡危险；而不是说如果事件严重时会导致死亡。

7.重要不良事件

除严重不良事件外，发生的任何导致采用针对性医疗措施（如停药、降低剂量和对症治疗）的不良事件和血液学或其他实验室检查明显异常。

注释：判定某一事件是否为重要的医学事件时应进行医学及科学的判断。某一重要的医学事件可能不一定会立即危及生命和/或导致死亡或住院。但是如果确定某事件可能会危害受试者或可能需要医疗干预以预防上述任何一种严重不良事件结局的发生，那么该重要的医学事件应报告为严重不良事件。

8.不良事件的记录要求

自受试者签署知情同意书（ICF）至研究结束，无论受试者是否接受干细胞制剂治疗，无论是研究者观察到的还是受试者报告的不良事件均应记录于《不良事件报告表》，见备案资料十三号，研究者在研究期间应如实填写。AE 的种类、程度、出现时间、持续时间、处理措施、处理经过详细记录，在综合考虑合并症、合并用药的基础上，评价其与干细胞制剂的相关性。

临床研究方案要求的所有体格检查与实验室检查结果均应记录在受试者的原始病历中。将受试者给药治疗后的体格检查结果、实验室检查与给药治疗前的检查结果进行比较，若其改变提示受试者临床状态恶化，研究者必须进行评估，以判定它是否符合 AE 的定义，并将被确定符合 AE 定义的所有改变记录于原始病历本的不良事件部分。

有关不良事件的医学文件均应记录在原始文件中，包括肺功能检查、实验室检查、影像学 CT 检查的检查通知单和检查结果报告单等。如受试者因研究结束或受试者出院等而无法继续接受研究者的治疗，研究者应将受试者的病历摘要（包括治疗安排和不良事件是否需要继续随访的说明等）交给负责继续治疗他们的医生。这些信息也要记录在原始文件中。

AE 需由研究者随访至消失/基线水平或得知最终结果。

9.不良事件的报告

9.1 预期不良事件：及时进行收集、记录，定期报告给伦理委员会。

9.2 非预期不良事件：及时进行收集、记录，定期报告给伦理委员会，评估风险，研究者协助制剂制备机构写出安全性报告交机构伦理委员会，并通报所有研究者，必要时对方案及知情同意书进行修正，同时修改研究者手册。

9.3 严重不良事件：如发生任何严重不良事件或重要的不良事件，无论是否与研究干预有关，也无论是否已实施干预操作，研究者均应在获知后 24 小时内向机构、伦理委员会、制剂制备机构、湖北省和国家卫生健康委员会报告，临床研究机构保证满足所有法律法规要求的报告程序。研究者应在原始资料中应记录何时、以何种方式、向谁报告了严重不良事件。

严重不良事件联系人列表如下：

姓名	职能	固定电话	手机
总值班	医院业务总值班		15327281120
叶青松	主要研究者	027-88041911-86346	15858242516
周晨亮	主要研究者	027-88041911-83920	18171234829
程丽薇	机构办公室	027-88041911-86346	18971566872
黄珍	伦理委员会	027-88041911-81319	
沈波	医务处	027-88041911-82285	13971158718
贺嘉	制剂制备机构	010-83602352-807	18910765084
-	湖北卫健委	027-87576368	
尹旭珂	国家卫健委	010-68792955	

研究者必须填写“SAE 报告表”，所有研究相关信息必须记录在 SAE 表，包括对严重不良事件临床过程的描述、评估严重程度、与研究药物或研究程序的因果关系、采取措施及事件结束的日期。受试者应被密切观察直至情况消失，或者病因已经确定并且所有随访信息均已记录在严重不良事件随访报告表中，。

发生 SAE 时研究者均应给予及时抢救处理，出现 SAE 退出本研究的受试者将

被继续随访至正常或有合理医学解释为止。随访方式可以根据不良事件的轻重选择住院、门诊、家访、电话等形式。

10.研究期间妊娠事件的处理与报告

绝经期前的女性受试者一旦在研究随访期间发生妊娠事件，研究者应当立即中止该受试者临床研究。研究者应根据干细胞制剂信息科学、严谨地与受试者交流，告知其干细胞制剂对于孕妇及胎儿可能的影响和风险。在确认育龄女性受试者发生妊娠事件的 24 小时内，研究者应当填写“妊娠报告表”，见备案资料十三号，并报告机构、伦理委员会、制剂制备机构、湖北省和国家卫生健康委员会。

（十七）研究的风险与受益

1.风险及防范措施、处理预案：

1.1 心理方面的风险及处理：部分受试者可能会因为对干细胞制剂的认识不足而产生心理上的压力。若有，研究者给与心理干预，详细介绍目前国内与国际上干细胞临床研究的现状，本研究中干细胞制剂可能的作用机制，以及临床出现预期不良事件后的处理，并告知受试者可以在任意时间无理由退出，消除或缓解受试者的心理压力。

1.2 社会方面的风险及处理：部分受试者可能会对本研究过程中采集的个人信息的安全性产生担忧。若有，研究者向受试者说明，在研究中受试者的私人信息在任何时候都将被严格保密，并告知受试者本研究所采用的保密措施。同时说明，受试者的私人信息仅限于在必要时提供给本项目的研究者、伦理委员会以及国家药品监督管理部门。

1.3 经济方面的风险及处理：受试者可能会对研究过程中的花费产生疑虑，以及对参加本研究引起的误工费有负担。若有，研究者向受试者申明在本研究中因参加本研究而进行的常规治疗、干细胞输注治疗和肺功能检查、实验室检查、影像学检查（CT）的费用承担情况。同时，研究者申明受试者参加本研究将获得每次300 元的费用作为参加本研究随访访视的交通补助和误工补偿，由研究者负责在随访访视结束后以现金方式发放给受试者。

2.受益：

2.1 受试者的受益：本研究拟入组新型冠状病毒所致重症肺炎的受试者。研究过程中，如果受试者被分配到干细胞治疗组，经常规治疗联合干细胞输注治疗后，

受试者新型冠状病毒感染所致的重症肺炎治疗情况有可能进一步改善。

2.2 科学和社会的受益：对于新型冠状病毒所致重症肺炎，目前的常规治疗方法仍不能完全有效，存在一定死亡率。本研究旨在评价应用人牙髓间充质干细胞治疗新型冠状病毒所致重症肺炎的安全性和有效性；为探索应用人牙髓间充质干细胞治疗新型冠状病毒所致重症肺炎，降低死亡率，改善临床预后提供新的治疗方案。

（十八）研究结果的统计分析

1.数据管理

本研究采用医院电子病历进行数据采集，由研究者按照 GCP 和研究方案要求准确、及时、真实、完整、规范地记录填写电子病历，签字并注明日期。研究者同时填写病例报告表（CRF），所有栏目必须填写，不得随意更改，确实需要更改时，在更改的地方划一横线，保留原始数据，填入更改后数据签名并注明更改日期。所有的文字记录不带出病房，而是由电子版的方式传出，然后打印出来建档双备案（打印版和电子版）。完成的 CRF 由项目组质控人员审查后，第一联移交统计分析人员，进行数据录入与管理工作。数据管理采用 Epidata 软件，由统计分析人员独立进行双份录入并校对。对 CRF 中存在的疑问，统计分析人员将向研究者发出询问，根据研究者的回答进行数据修改、确认与录入，必要时统计分析人员可以再次发出询问。在数据审核完成后，由主要研究者、统计分析人员和合作的制剂制备机构共同对数据进行审核，并完成分析人群的最后定义及判断，之后由统计分析人员对数据库进行锁定。

2.统计分析

本项研究的统计分析人员不参与筛选、纳入受试者和后续的临床研究。研究过程中，仅对统计分析人员保持盲态。

（1）分析人群：

研究结果的统计分析数据集包括全分析数据集（FAS）、符合方案数据集（PPS）、安全性数据集（SS）。

全分析集（FAS，Full Analysis Set）：对所有入组后已使用研究药物并至少完成一次疗效评估的病例。对主要指标缺失值的估计，采用末次观察值结转（LOCF，last observation carrying forward）的方法；

符合方案集（PPS，Per Protocol Set）：是 FAS 的子集，所有符合研究方案、

依从性好、研究期间未用禁止用药、完成整个临床观察并完成 CRF 规定填写内容的病例；

安全性分析集（SS，Safety Set）：所有入组后接受研究药物并至少有一次安全性评估的病例。

本研究中，基线资料的分析采用 FAS 分析。主要疗效指标、次要疗效指标等同时进行 FAS 和 PPS 分析。但以 FAS 分析所得结论为主。当 FAS 和 PPS 所得结论一致时，可以增加结论的可信度。

安全性分析中，不良事件和不良反应资料、生命体征、实验室指标的分析采用 SS。由于不良事件及各种非治疗原因提前终止的受试者，计入安全性分析。

（2）统计分析计划：

研究结果分析内容包括受试者分布描述、基线指标的均衡性分析、疗效分析、安全性分析。

计量资料的统计描述采用均数、标准差、中位数、四分位数、最小值和最大值，组间比较采用方差分析或 Kruskal-Wallis 秩和检验，组内前后比较采用配对 t 检验或 Wilcoxon 符号秩检验。

计数与等级资料的统计描述采用率或构成比，计数资料的比较采用 x^2 检验或 Fisher 精确概率法，等级资料的比较采用 Kruskal-Wallis 秩和检验。

（十九）研究的质量控制与保证

1.质量控制措施

制剂制备机构及研究者均应采用标准操作规程，以保证临床研究的质量控制和质量保证系统的实施。临床研究中所有观察结果和发现都应加以核实，以保证研究数据的可靠性，确保临床研究中各项结论来源于原始数据。在数据处理的每一阶段必须采用质量控制，以保证所有数据可靠，处理正确。

2.研究者的培训

临床研究开始前，研究项目负责人应对研究者进行研究方案的培训，以让研究者了解并熟悉干细胞制剂的性质、作用、疗效及安全性（包括干细胞制剂的临床前研究资料和前期临床研究资料），同时也应掌握临床研究进行期间发现的所有与干细胞制剂有关的新信息。

3.提高受试者的依从性

受试者取得随机分配号并分配给本研究项目的研究者后，由研究者负责受试者的跟踪随访。研究者应认真执行赫尔辛基宣言、中国现行法规，以及本临床研究方案和知情同意书，使受试者充分理解研究要求，配合研究，并定期电话提醒受试者随访时间。

4.伦理学要求和知情同意

本研究遵循《赫尔辛基宣言》所阐述的准则，遵守《药物临床试验质量管理规范》（2003年）、《干细胞临床研究管理办法》（试行，2015年）、《干细胞制剂质量控制及临床前研究指导原则（试行，2015年）等相关规定。研究方案及其修订、知情同意书及其修订、病例报告表（CRF）及其修订和需提供给受试者的其他书面资料均须经过武汉大学人民医院临床研究伦理委员会批准后实施。

研究者负责向每个受试者解释参加本研究的受益和风险，受试者在签署知情同意书（ICF）后方能参加本研究。最终的知情同意书文本应包含以下内容：研究目的、研究步骤、受试者的义务、参加该研究可能带来的可预见的益处及可预见的风险与不便；发生与研究相关的损害时，受试者可以获得的治疗和适当的补偿；研究资料的查阅及受试者信息的保密。

知情同意书应以受试者可阅读的语言写成。受试者、执行知情同意过程的研究者均需在知情同意书上签名并注明日期。知情同意书一式两份，分别由研究机构和受试者保存。

当受试者没有能力、或不能充分地给予知情同意时（如精神障碍者），应获得合法代理人的知情同意；同时，应根据受试者可理解程度告知受试者有关研究的情况；如可能，受试者应签署书面知情同意并注明日期。

在研究过程中，如发现涉及干细胞制剂新的重要信息则必须将知情同意书作书面修改，并经武汉大学人民医院临床研究伦理委员会批准后，再次取得受试者同意。

5.研究方案的变更

本研究方案经武汉大学人民医院临床研究伦理委员会批准后，若做出任何修改，应由研究者与制剂制备机构协商后决定，并写出“临床研究方案修改说明”及“临床研究方案修改对照表”，由主要研究者签字，需再次获得武汉大学人民医院临床研究伦理委员会批准方可实施。任何参加研究的人员不得违背本研究方案。

6.各方职责

（1）研究者

研究者应保障所实施临床研究的质量，保护受试者安全和权益。临床研究机构应建立与临床研究相适应的组织架构，建立相应的管理体系并配备相关人员、设施设备，对机构承担的干细胞临床研究进行管理。

研究者应向受试者说明经武汉大学人民医院临床研究伦理委员会同意的有关干细胞临床研究的详细情况，并在临床研究开始前取得知情同意书，相关检查治疗费用由课题经费和武汉大学人民医院或合作单位支付。

（2）制剂制备机构

制剂制备机构负责发起、申请、组织、监查干细胞临床研究，并根据临床研究项目合作协议提供制剂制备所需经费。

研究者违背已批准的研究方案或有关法规进行干细胞临床研究时，制剂制备机构应指出以求纠正，如情况严重或坚持不改，则应终止研究者参加临床研究并向武汉大学人民医院临床研究伦理委员会、卫健委和食品药品监督管理部门报告。

7.资料保存

为保证国家卫健委和国家药品监督管理局的监督管理，临床研究机构应按照国家有关法规的规定保存所有本临床研究项目的资料，包括对所有受试者的确认（能有效地核对不同的记录资料，如受试者的原始病历本和病例报告表等），所有原始有签名的受试者知情同意书，所有受试者原始病历本、病例报告表等，以及临床研究方案、研究者手册、学术委员会批件、伦理委员会批件、研究协议、干细胞制剂分发的详细记录、不良事件及严重不良事件记录等的原件，同时制剂制备机构保留一份复印件。所有记录保存期按国家药政管理部门的要求执行。

（二十）知情同意书（见备案资料十六号文件）

（二十一）附件

附件 1：研究访视与检查表

附件 1：

研究访视与检查表

<table>
<tr><th rowspan="3">序号</th><th rowspan="3" colspan="2">访视项目</th><th colspan="8">访 视 时 间 与 次 序</th></tr>
<tr><th>筛选期</th><th colspan="6">治疗与观察期（D1-D28 或出院）</th><th>随访期</th></tr>
<tr><th>-D7 天</th><th>D1</th><th>D4</th><th>D7</th><th>D10</th><th>D14</th><th>D28 或出院日</th><th>D90（±3D）</th></tr>
<tr><td>1</td><td colspan="2">签署知情同意书，分配筛选号</td><td>X</td><td></td><td></td><td></td><td></td><td></td><td></td><td></td></tr>
<tr><td>2</td><td colspan="2">入、排标准</td><td>X</td><td></td><td></td><td></td><td></td><td></td><td></td><td></td></tr>
<tr><td>3</td><td colspan="2">人口统计学资料（性别、出生日期、民族、身高、体重）</td><td>X</td><td></td><td></td><td></td><td></td><td></td><td></td><td></td></tr>
<tr><td>4</td><td colspan="2">病史资料</td><td>X</td><td></td><td></td><td></td><td></td><td></td><td></td><td></td></tr>
<tr><td rowspan="2">5</td><td rowspan="2" colspan="2">生命体征：血压、心率</td><td rowspan="2">X</td><td colspan="6">X 住院期间每日进行</td><td rowspan="2">X</td></tr>
<tr><td>X（输注前 30min 和输注结束后 2h±30min、24h±30min）</td><td>X（输注前 30min 和输注结束后 2h±30min、24h±30min）</td><td>X（输注前 30min 和输注结束后 2h±30min、24h±30min）</td><td>X</td><td>X</td><td>X</td></tr>
<tr><td rowspan="2">6</td><td rowspan="2" colspan="2">临床检测指标（血氧饱和度、呼吸频率、咳嗽程度测量值、体温指标）</td><td rowspan="2">X</td><td colspan="6">X 住院期间每日进行，直至指标恢复</td><td rowspan="2"></td></tr>
<tr><td>X（输注前 30min 和输注结束后 2h±30min、24h±30min）</td><td>X（输注前 30min 和输注结束后 2h±30min、24h±30min）</td><td>X（输注前 30min 和输注结束后 2h±30min、24h±30min）</td><td>X</td><td>X</td><td>X</td></tr>
<tr><td>7</td><td colspan="2">临床改善时间（TTCI）时间范围</td><td></td><td colspan="6">X 住院期间每日进行，直至六类有序等级变量下降两个等级</td><td></td></tr>
<tr><td>8</td><td>实验室</td><td>血常规</td><td>X</td><td>X（输注前 30min 和输注结束后 2h±30min、24h±30min）</td><td>X（输注前 30min 和输注结束后 2h±30min、24h±30min）</td><td>X（输注前 30min 和输注结束后 2h±30min、24h±30min）</td><td>X</td><td>X</td><td>X</td><td>X</td></tr>
</table>

9	检查	肝肾功	X	X(输注前 30min 和输注结束后 2h±30min、24h±30min)	X（输注前 30min 和输注结束后 2h±30min、24h±30min）	X（输注前 30min 和输注结束后 2h±30min、24h±30min）	X	X	X	X
10		传染病检测：乙肝、丙肝、艾滋、梅毒、巨噬细胞病毒-IgM、巨噬细胞病毒-IgG	X							X
11		炎症检查（超敏 C 反应蛋白、血清淀粉样蛋白 SAA）	X	X(输注前 30min 和输注结束后 2h±30min、24h±30min)	X（输注前 30min 和输注结束后 2h±30min、24h±30min）	X（输注前 30min 和输注结束后 2h±30min、24h±30min）	X	X	X	X
12		尿常规	X	X（输注前 30min 和输注结束后 2h±30min、24h±30min）	X（输注前 30min 和输注结束后 2h±30min、24h±30min）	X（输注前 30min 和输注结束后 2h±30min、24h±30min）	X	X	X	X
13		便常规	X	X(输注前 30min 和输注结束后 2h±30min、24h±30min)	X（输注前 30min 和输注结束后 2h±30min、24h±30min）	X（输注前 30min 和输注结束后 2h±30min、24h±30min）	X	X	X	
14		免疫学检查：淋巴细胞亚群 CD3、CD4、CD8、CD19、CD16、CD56 细胞计数；IgA、IgG、IgM、总 IgE，IL-1β，IL- 2、IL- 4、IL- 6、IL- 10、TNF-a,、ITN-γ	X	X（输注前 30min 和输注结束后 2h±30min、24h±30min）	X（输注前 30min 和输注结束后 2h±30min、24h±30min）	X（输注前 30min 和输注结束后 2h±30min、24h±30min）	X	X	X	X
15		妊娠检查	X							X
16		新冠状病毒检测	X	X	X	X		X	X<br>出院前<br>连续 2 次	
17		胸部 CT	X	X		X		X	X	
18		记录合并用药及合并治疗情况	X	X	X	X	X	X	X	X
19		记录 AE 和 SAE		X	X	X	X	X	X	X
20		日记卡的发放和回收	X						X	X
21		受试者信息卡的发放	X	X					X	
